# Ultrathin 2D metal–organic framework nanosheets prepared *via* sonication exfoliation of membranes from interfacial growth and exhibition of enhanced catalytic activity by their gold nanocomposites

**DOI:** 10.1039/c9ra00662a

**Published:** 2019-03-25

**Authors:** Songting Wu, Lu Qin, Ke Zhang, Zhong Xin, Shicheng Zhao

**Affiliations:** Shanghai Key Laboratory of Multiphase Materials, School of Chemical Engineering, East China University of Science and Technology Shanghai 200237 China zhaosc@ecust.edu.cn

## Abstract

Ultrathin two-dimensional (2D) metal–organic framework (MOF) nanosheets were prepared by a facile sonication exfoliation of MOF membranes from interfacial growth. The stacked form of nanosheets constituting the MOF membranes was significantly different to that of its layered MOF counterparts. This led to decreased interaction between nanosheets, so they could exfoliate readily from the MOF membranes. Moreover, Au nanoparticles were introduced to form nanocomposites. Enhanced catalytic activity and long-term stability of these nanocomposites were observed by a model reaction of the reduction of 4-nitrophenol to 4-aminophenol. This preparation method could be extended to other 2D MOF nanosheets and their nanocomposites.

## Abstract 2

Cu-MOF nanosheets were prepared by sonication exfoliation and the Au/Cu-MOF nanocomposites exhibited higher catalytic activity than pure Au NPs.

## Graphical abstract

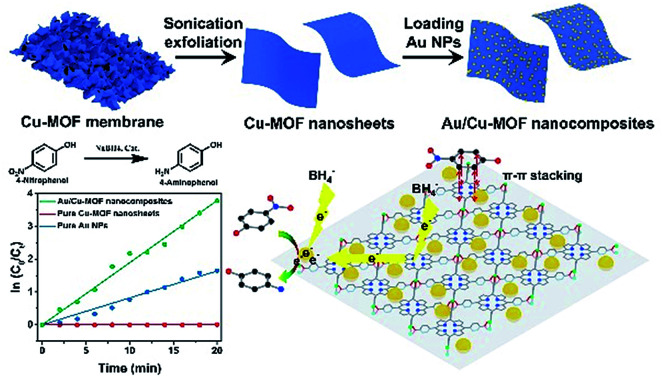

## 1. Introduction

Since graphene was exfoliated from graphite by mechanical cleavage in 2004,^1,2^ this two-dimensional (2D) nanomaterial with unique structure as well as intriguing physical and chemical properties has garnered considerable interest. Up to now, several graphene analogs, such as graphene oxide,^3^ hexagonal boron nitride,^4^ transition metal dichalcogenides,^5^ graphitic carbon nitride,^6^ layered double hydroxides,^7^ noble metal nanosheets,^8–10^ covalent-organic frameworks,^11^ and 2D planar hypercoordinate materials (*e.g.* AlB_6_, Cu_2_Si)^12–17^ have enriched the “family” of 2D nanomaterials.^18,19^ These layered nanomaterials exhibit many unique properties compared with zero-dimensional (0D) nanoparticles, one-dimensional (1D) nanowires, three-dimensional (3D) networks, and their bulk counterparts,^18,20–22^ which have attracted considerable efforts for exploration of other 2D nanomaterials. Recently, 2D metal–organic framework (MOF) nanosheets, a new class of crystalline porous materials constructed by linking metal nodes (*e.g.*, metal ions, clusters) with organic ligands (*e.g.*, carboxylate ligands, other negatively charged ligands),^23^ have attracted increasing research interest. Meanwhile, the power of multi-scale computational simulation has led to rapid development in the experimental synthesis and application of MOFs^24–31^ to deepen our understanding of MOF nanosheets. Their unique features, such as tunable structure and function, abundant active sites, extended lateral dimension, and ultrahigh porosity,^32,33^ make them promising candidates for application in catalysis,^34–36^ electrochemistry,^37^ gas separation,^38,39^ sensing,^40^ and other applications.^32^

Strategies for the synthesis of 2D MOF nanosheets can be classified into two categories: bottom-up and top-down. The former refers to the direct synthesis of 2D MOF nanosheets from metal salts or metal sources and organic linkers, whereas the latter strategy involves the exfoliation of bulk-layered MOF counterparts. Recently, bottom-up methods have been developed extensively, such as surfactant-assisted synthesis,^41,42^ modulated synthesis,^43,44^ three-layer synthesis,^39^ direct synthesis^45,46^ and layer-by-layer methods.^39^ However, selectively blocking growth in the vertical direction without affecting the lateral dimension, while keeping good control of the structure, thickness, and crystallinity of the deposited material, remains a great challenge.^32,47^ In contrast, the top-down method has been demonstrated to be a formidable approach for efficient and scalable production of various 2D MOF nanosheets.^48,49^ However, the van der Waals forces or hydrogen bonding between adjacent layers make it difficult to achieve complete exfoliation.^20,49^ Even though Li-intercalation^50^ and chemical exfoliation^51^ have been developed to prepare ultrathin 2D MOF nanosheets effectively, the subsequent removal of intercalators would increase the production cost, which affects the large-scale fabrication of 2D MOF nanosheets. Therefore, achieving facile and additive-free synthesis of 2D MOF by top-down methods remains a major challenge.

In addition, MOFs themselves may not meet specific application needs,^52^ such as cascade reactions,^53,54^ and selective sensing.^55^ As demonstrated recently, 2D MOF nanosheets have been utilized as burgeoning supports to disperse and stabilize metals,^53,56–58^ metal oxide nanoparticles (NPs)^59^ and metal sulfides,^60^ which not only avoid the aggregation of free metal NPs with high surface energies, but also greatly improve their activities and even generate a synergetic effect between them, thus leading to improved and/or some unattainable performance compared with the individual components.^53,57^ Herein, we demonstrate a facile strategy for preparation of ultrathin MOF nanosheets *via* sonication exfoliation of 2D MOF membranes from interfacial growth. Our results showed that the stacked form of the nanosheets constituting the membrane was significantly different with that of its layered bulk MOF counterparts, which may have decreased the interaction between nanosheets and made exfoliation much easier. Then, Au NPs were decorated on the as-prepared 2D MOF nanosheets by *in situ* growth. As proof of the structural advantages, Au/Cu-MOF nanocomposites were used as a catalyst, and their catalytic activity evaluated by a model reaction of the reduction of 4-nitrophenol (4-NP) to 4-aminophenol (4-AP) with sodium borohydride (NaBH_4_). Impressively, a synergistic effect was observed between Au NPs and Cu-MOF nanosheets.

## 2. Experimental section

### 2.1 Materials

All chemicals were purchased from commercial suppliers without further purification unless mentioned otherwise. Tetrakis(4-carboxyphenyl)porphyrin (TCPP, 97%), 1-octanol (99%) and copper acetate monohydrate (Cu(CH_3_COO)_2_·H_2_O, 99%) were purchased from TCI. Hydrogen tetrachloroaurate trihydrate (HAuCl_4_·3H_2_O, 99.9%), sodium borohydride (99%), sodium citrate (99.8%), 4-nitrophenol (4-NP, 99%), and dimethylformamide (DMF, 99.8%) were purchased from Adamas.

### 2.2 Synthesis of 2D Cu-MOF nanosheets

Cu-MOF nanosheets were synthesized *via* sonication exfoliation of Cu-MOF membranes from interfacial growth. Briefly, the aqueous phase was prepared by dissolving Cu(CH_3_COO)_2_·H_2_O (5 wt%) in water. TCPP (0.12 wt%) was dissolved in a mixed solution of 1-octanol and DMF (v/v, 9 : 1), as the organic phase. Then, the organic phase (10 mL) was layered on the aqueous phase (10 mL) in a 30 mL vial for 3 days. The product at the interface of the two immiscible solvents could be “dug out” by a spoon. After washing membranes thrice with ethanol freeze-drying, Cu-MOF nanosheets (0.2 mg mL^−1^) were obtained by sonication exfoliation of membranes in water for 20 min.

### 2.3 Synthesis of Au/Cu-MOF nanocomposites

Au/Cu-MOF nanocomposites were prepared *via* a facile one-pot surfactant-free method. Briefly, HAuCl_4_ (0.05 wt%), Cu-MOF nanosheets (0.1 mg mL^−1^) and sodium citrate (0.05 wt%) were mixed with sonication for 2 min and then placed in an oil bath at 100 °C for 5 min with stirring. After washing by centrifugation until the supernatant was colorless, the resulting Au/Cu-MOF nanocomposites (0.1 mg mL^−1^) were re-dispersed in water prior to use.

### 2.4 Synthesis of Au NPs

Au NPs were synthesized as reported previously.^61^ Briefly, a solution of HAuCl_4_ (10 mL, 0.05 wt%) was brought to boil under stirring. A solution of sodium citrate (0.1 mL, 5 wt%) was added rapidly. After boiling of the solution for 5 min, a brilliant red solution was obtained. Finally, the solution was stirred at room temperature. The resulting Au NPs were washed by centrifugation and re-dispersed in water prior to use.

### 2.5 Characterization

Powder X-ray diffraction (PXRD) patterns were measured by a rotating anode X-ray powder diffractometer (Rigaku) using Cu Kα radiation (*λ* = 1.54178 Å). Field-emission scanning electron microscopy (FESEM) images were obtained from a JSM-6360LV microscope (JEOL). Transmission electron microscopy (TEM) images, high-resolution TEM (HRTEM) images and selected area electron diffraction (SAED) images were taken on a JEM-1400 (JEOL) microscope operating at an acceleration voltage of 100 kV. Atomic force microscopy (AFM) using a DI system (Veeco Instruments) was used to characterize nanosheet thickness. The UV-vis absorption spectra of samples were collected on a Lambda 950 spectrophotometer (PerkinElmer) with QS-grade quartz cuvettes (110-QS; Shimadzu).

Prior to characterization by TEM and AFM, the nanosheets were obtained from Cu-MOF membranes by ultrasound treatment in ethanol. Then, the ethanolic suspension was dropped onto holey, carbon-coated, carbon-supported copper grids and piranha-cleaned Si, respectively, and then dried naturally.

## 3. Results and discussion

### 3.1 Characterization of Cu-MOF nanosheets

**Fig. 1:**
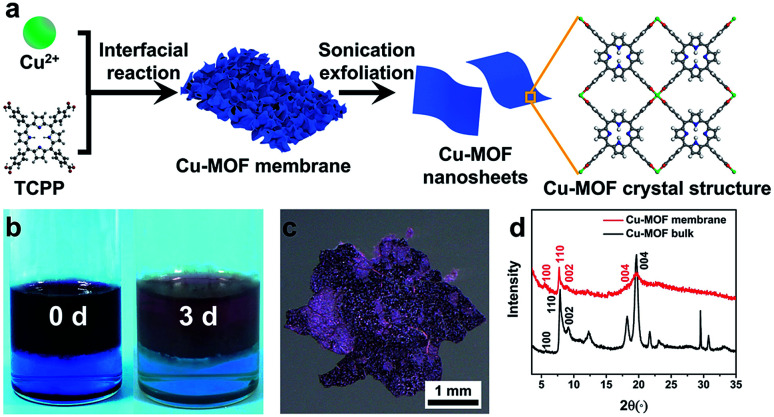
(a) Synthetic procedure and crystal structure of Cu-MOF nanosheets (schematic). (b) Photographs of the color changes of two immiscible phases before and after the reaction. (c) Optical image of a Cu-MOF membrane. (d) PXRD spectra of a Cu-MOF membrane and Cu-MOF bulk counterparts.

Cu-MOF membranes were initially synthesized *via* interfacial growth by layering the organic phase on the aqueous phase, then Cu-MOF nanosheets were obtained through sonication exfoliation of Cu-MOF membranes (Fig. 1a). Crystals of Cu-MOF nanosheets containing TCPP ligands were linked by four Cu_2_(COO)_4_ “paddlewheel” metal nodes to form a “checkerboard”-like layered structure.^62^ As expected, discoloration of the organic and aqueous phase was observed after 3 days (Fig. 1b), which meant that TCPP and metal ions had reacted at the interface of two immiscible solvents. The large size and free-standing Cu-MOF membranes formed at the interface were transferred to a glass slide and a claret-colored membrane with a rough surface was observed by optical microscopy (Fig. 1c). The PXRD spectrum of Cu-MOF membranes gave four typical peaks (100), (110), (002) and (004) respectively, which matched well with the bulk MOF counterparts (Fig. 1d), thereby indicating their similar crystal structure.^62,63^

**Fig. 2:**
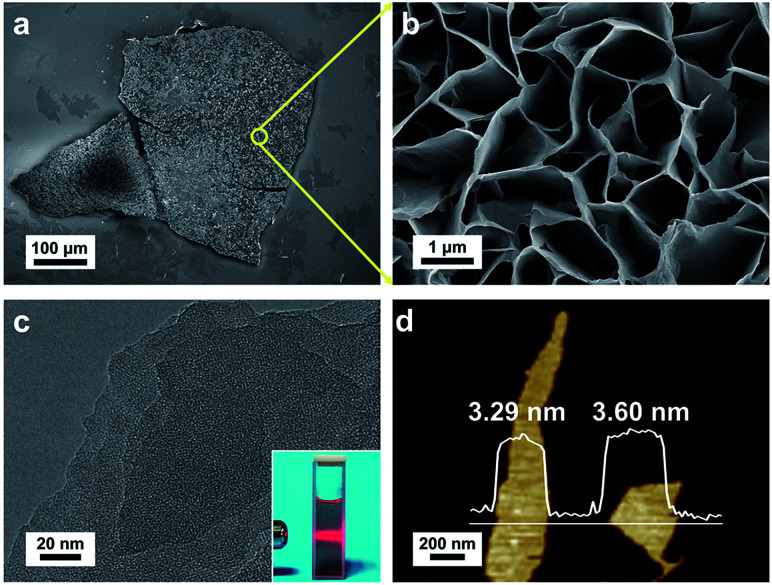
(a) FESEM image of the overview of a Cu-MOF membrane. (b) High-magnification FESEM image of the surface of a Cu-MOF membrane. (c) TEM image of Cu-MOF nanosheets, inset: the Tyndall effect of a colloidal suspension. (d) AFM image of Cu-MOF nanosheets.

FESEM was used to characterize the morphology of the synthesized Cu-MOF membranes. The FESEM image of Cu-MOF membranes displayed a rough surface (Fig. 2a). Surprisingly, the high-magnification FESEM image of the surface of Cu-MOF membranes showed numerous nanosheets interlaced with each other, which were very different from the traditional bulk 2D MOF counterparts with stacked layers (Fig. 2b). This stacked form decreased the interaction between nanosheets, which could lead to these membranes being exfoliated readily to ultrathin nanosheets by sonication. Then, the as-prepared Cu-MOF membranes were exfoliated to ultrathin nanosheets in water within 20 min. The MOF nanosheets had good dispersion and stability in water. Exfoliation was evidenced by the Tyndall effect upon irradiation with a laser beam (inset in Fig. 2c). The exfoliated nanosheets were characterized by TEM and AFM. Both characterization methods indicated that free-standing nanosheets with thickness of nanometers were obtained after exfoliation (Fig. 2c and d). The low contrast of MOF nanosheets in TEM images showed their ultrathin nature (Fig. 2c). The AFM image of MOF nanosheets showed a uniform thickness of ∼3.4 nm (Fig. 2d), and the layer number of obtained nanosheets was ∼4 according to reported crystallographic data.^62^ The data mentioned above revealed successful preparation of ultrathin Cu-MOF nanosheets by sonication exfoliation of MOF membranes from interfacial growth.

### 3.2 Characterization of Au/Cu-MOF nanocomposites

**Fig. 3:**
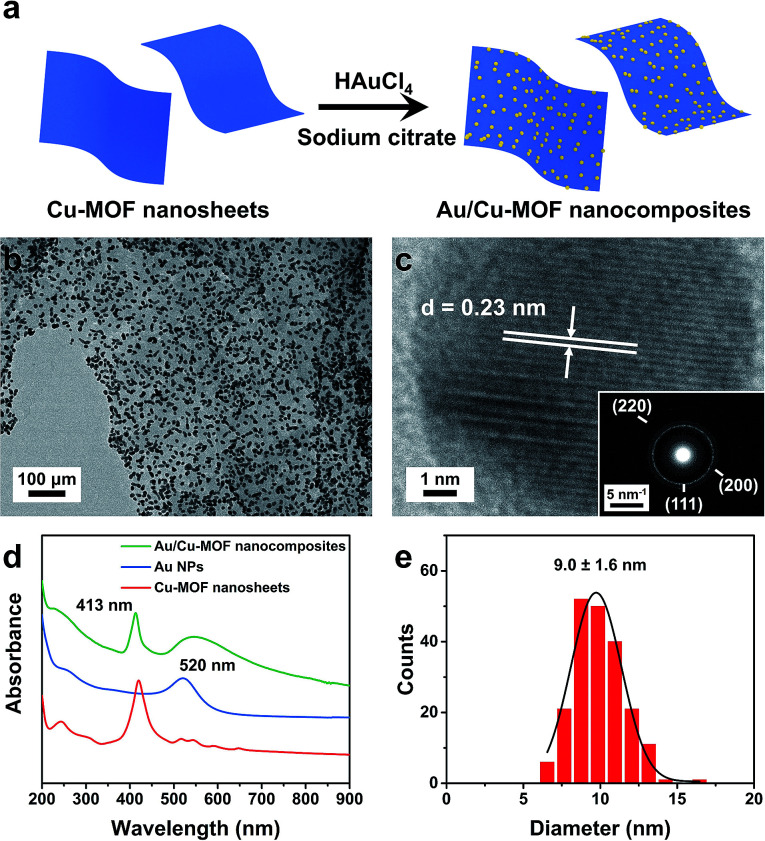
(a) Synthesis of Au/Cu-MOF nanocomposites (schematic). (b) TEM image of Au/Cu-MOF nanocomposites. (c) HRTEM image of Au NPs on nanosheets, inset: the corresponding electron diffraction pattern. (d) UV-vis absorption spectra of Cu-MOF nanosheets, Au NPs, and Au/Cu-MOF nanosheets dispersed in water, respectively. (e) Size-distribution histogram of Au/Cu-MOF nanocomposites.

Au/Cu-MOF nanocomposites were prepared *via* a facile one-pot surfactant-free method by *in situ* hydrothermal growth of Au NPs on nanosheets (Fig. 3a). The obtained Au/Cu-MOF nanocomposites were characterized by TEM and UV-vis spectroscopy. The TEM image of Au/Cu-MOF nanocomposites showed that numerous Au NPs were attached homogeneously to the MOF nanosheets, and that the nanosheets were not damaged, indicating their excellent water stability and thermal stability (Fig. 3b). The HRTEM image of Au NPs revealed a lattice spacing of 0.23 nm, corresponding to the (111) plane (Fig. 3c). The corresponding SAED pattern further confirmed the high crystallinity of Au NPs (inset in Fig. 3c). In addition, the UV-vis spectra of pure Cu-MOF nanosheets showed a strong Soret band at 414 nm and four Q-bands in the range 500–700 nm (Fig. 3d, red curve).^62^ After decorating Au NPs on Cu-MOF nanosheets, the four Q-bands were covered by a wide-ranging peak (Fig. 3d, green curve), which could be ascribed to the characteristic surface plasmon absorption peak for the Au NPs^64^ (Fig. 3d, blue curve). The size distribution of Au NPs showed a uniform diameter of 9.0 ± 1.6 nm (Fig. 3e). Due to the complete reduction of Au NPs by sodium citrate, the mass ratio of Cu-MOF nanosheets : Au NPs in the resulting nanocomposites was estimated to be 1 : 2.9. Taken together, these results revealed that Au/Cu-MOF nanocomposites were prepared.

### 3.3 Catalytic activity of Au/Cu-MOF nanocomposites

**Fig. 4:**
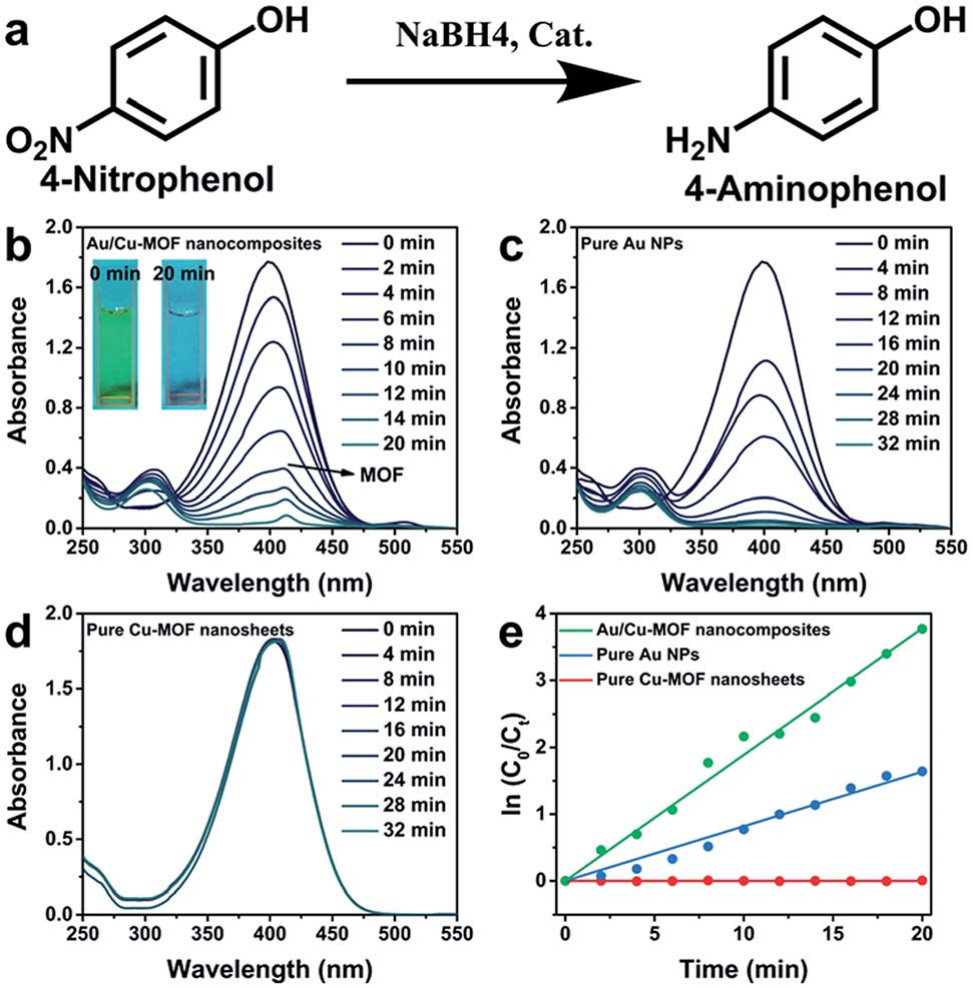
(a) The model reaction of the reduction of 4-NP to 4-AP with NaBH_4_. Time-dependent UV-vis absorption spectra of the reduction reaction process with (b) Au/Cu-MOF nanocomposites, (c) pure Au NPs, and (d) pure Cu-MOF nanosheets as catalyst, respectively. Inset in (b): changes in color of the mixture solution before and after the reaction. (e) Relationship between ln(*C*_t_/*C*_0_) and reaction time (*t*) of Au/Cu-MOF nanocomposites, pure Au NPs and pure Cu-MOF nanosheets, respectively.

Various 3D MOF crystal-encapsulated Au NPs nanocomposites with enhanced catalytic activity have been reported.^65–68^ 3D MOF crystals are microporous materials that can provide selectivity in a multi-reactant reaction but may also confine the diffusion of reactants in a single-reactant reaction.^67,69^ In this work, because Au NPs were decorated on the surface of nanosheets, the diffusion of reactants did not occur in Au/nanosheet composites. To demonstrate the structural advantages of Au/Cu-MOF nanocomposites, the model reaction of 4-NP reduction with NaBH_4_ in water (Fig. 4a), a well-known reaction that cannot proceed without catalysts but can proceed rapidly in the presence of metallic surfaces, was used to examine catalytic activity.^68,70^ Typically, a freshly prepared aqueous solution of NaBH_4_ (0.20 mL, 0.1 M) was mixed with an aqueous solution of 4-NP (2.8 mL, 0.1 mM) at room temperature. Then, Au/Cu-MOF nanocomposites (100 μL, 0.1 mg mL^−1^) were added to the mixture solution. The bright-yellow solution turned colorless rapidly (within 20 min) as observed by the naked eye (inset in Fig. 4b), thereby indicating the complete reduction of 4-NP to 4-AP. The UV-vis absorption spectra were recorded to monitor the progress of the reaction. As the absorption peak of 4-NP at 400 nm decreased, the characteristic peak of 4-AP at 300 nm increased concomitantly, and the inconspicuous absorption peak at 414 nm belonged to Cu-MOF nanosheets (Fig. 4b). A much longer reaction time was required over the same content of pure Au NPs (32 min) (100 μL, 0.029 wt%) (Fig. 4c) and pure Cu-MOF nanosheets (100 μL, 0.1 mg mL^−1^) were found to be catalytically inactive under identical conditions (Fig. 4d). This catalytic reduction could be considered to be a pseudo-first-order reaction.^70^ Accordingly, the rate constants using different catalysts could be calculated by the rate equation ln(*C*_0_/*C*_t_) = *k*_t_, where *k* is the kinetic rate constant, and *C*_0_ and *C*_t_ are the initial and apparent concentrations of 4-NP, respectively. Impressively, the rate constant of Au/Cu-MOF nanocomposites was estimated to be 0.200 min^−1^, which was nearly threefold higher than that of pure Au NPs (0.082 min^−1^) (Fig. 4e). This value is comparable with that of Au/graphene (0.1902 min^−1^).^71^

**Fig. 5:**
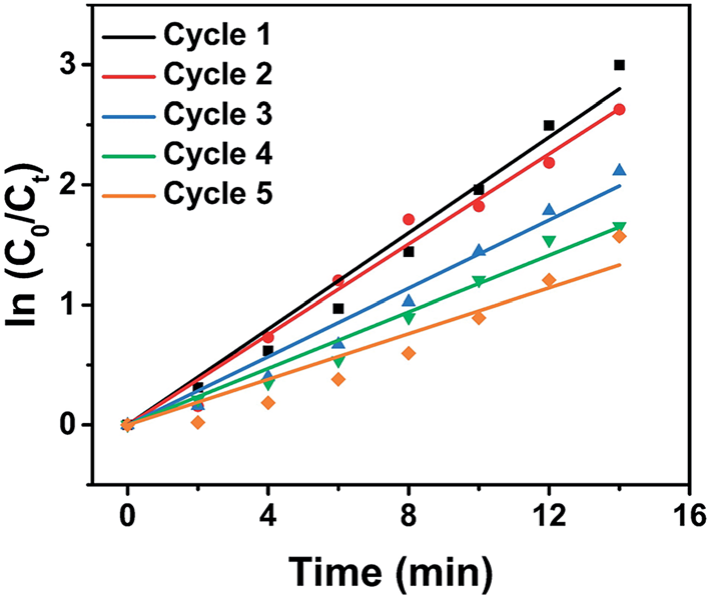
Relationship between ln(*C*_t_/*C*_0_) and reaction time (*t*) for five cycles of 4-NP reduction under identical reaction conditions over Au/Cu-MOF nanocomposites.

The recyclability of Au/Cu-MOF nanocomposites was also examined in the reduction of 4-NP. The nanocomposites were washed by centrifugation and reused for the next run under the same conditions after the reaction. As shown in Fig. 5 and Table 1, the *k* value decreased from 0.200 min^−1^ to 0.095 min^−1^ with an increase in cycle numbers, indicating that Au/Cu-MOF nanocomposites lost some catalytic activity after five cycles for the reduction reaction of 4-NP. However, this result was much better than that reported previously (decreases from 5.53 min^−1^ to 1.57 min^−1^).^67^ These results suggested that Au/Cu-MOF nanocomposites exhibit good stability.

**Table 1:** Recovery and reuse of Au/Cu-MOF nanocomposites under the same condition

Use	*k* (min^−1^)
First	0.200
Second	0.188
Third	0.142
Fourth	0.118
Fifth	0.095

### 3.4 Mechanism for the enhanced catalytic activity of Au/Cu-MOF nanocomposites

**Fig. 6:**
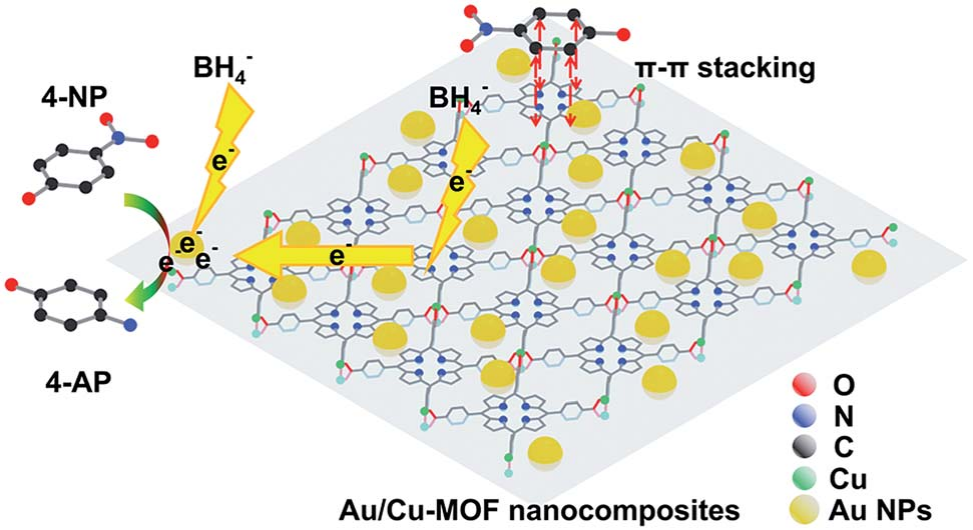
Proposed mechanism of the enhanced catalytic activity of Au/Cu-MOF nanocomposites for the reduction of 4-NP to 4-AP with NaBH_4_ (schematic). H atoms have been omitted for clarity.

Although pure Cu-MOF nanosheets were found to be catalytically inactive, Au/Cu-MOF nanocomposites exhibited higher catalytic activity than pure Au NPs. To understand this finding, one must distinguish the possible reasons for the enhanced catalytic activity of Au/Cu-MOF nanocomposites. From the results shown above, we proposed that the enhancement of catalytic activity could be ascribed to two factors: the interaction between Cu-MOF nanosheets and 4-NP, and the synergistic effect between Au NPs and Cu-MOF nanosheets. First, the 4-NP molecules could be adsorbed onto the surface of Cu-MOF nanosheets *via* π–π stacking interactions between the aromatic rings of 4-NP and TCPP,^66,71,72^ as well as the interactions between the hydroxyl group of 4-NP and the Lewis-acid active metal center in the nanosheets,^66^ which could increase the reaction probabilities between 4-NP and Au NPs. Conversely, the Au NPs decorated on Cu-MOF nanosheets, with enormous loading rates, high monodispersion, uniformity and small size compared with the Au NPs dispersed in solution, resulted in local high concentrations of catalytic active sites.^57,70,73^ Furthermore, the high electron mobility of 2D MOF nanosheets allowed electrons to be transferred rapidly from NaBH_4_ to the reactive sites, thereby speeding up the reaction.^71,73,74^ The possible mechanism for the enhanced catalytic activity of Au/Cu-MOF nanocomposites is illustrated schematically in Fig. 6.

## 4. Conclusion

We demonstrated a facile strategy for the synthesis of ultrathin 2D MOF nanosheets by sonication exfoliation of 2D MOF membranes from interfacial growth. A rough surface constituted by numerous nanosheets was observed on Cu-MOF membranes. The large space between nanosheets enabled exfoliation of Cu-MOF membranes by sonication. TEM and AFM images confirmed the uniform ultrathin properties of Cu-MOF nanosheets. The Tyndall effect also showed the stability of nanosheets dispersed in water. Moreover, Au NPs were decorated on 2D MOF nanosheets by *in situ* hydrothermal growth, and exhibited enhanced catalytic activity for the reduction of 4-NP and steady catalytic efficiency after several recycles. Finally, we also proposed a possible mechanism for the enhanced catalytic activity of Au/Cu-MOF nanocomposites. We believe that the developed materials will find more applications in catalysis, and that this preparation method could be extended to prepare other functional 2D MOF nanomaterials.

## Conflicts of interest

There are no conflicts of interest to declare.

## Supplementary Material
